# GSK-3 as potential target for therapeutic intervention in cancer

**DOI:** 10.18632/oncotarget.2037

**Published:** 2014-05-28

**Authors:** James A. McCubrey, Linda S. Steelman, Fred E. Bertrand, Nicole M. Davis, Melissa Sokolosky, Steve L. Abrams, Giuseppe Montalto, Antonino B. D'Assoro, Massimo Libra, Ferdinando Nicoletti, Roberta Maestro, Jorg Basecke, Dariusz Rakus, Agnieszka Gizak, Zoya Demidenko, Lucio Cocco, Alberto M. Martelli, Melchiorre Cervello

**Affiliations:** ^1^ Department of Microbiology and Immunology, Brody School of Medicine at East Carolina University Greenville, NC, USA; ^2^ Department of Oncology, Brody School of Medicine at East Carolina University Greenville, NC, USA; ^3^ Biomedical Department of Internal Medicine and Specialties, University of Palermo, Palermo, Italy; ^4^ Department of Medical Oncology, Mayo Clinic Cancer Center, Rochester, MN, USA; ^5^ Department of Bio-Medical Sciences, University of Catania, Catania, Italy; ^6^ Experimental Oncology 1, CRO IRCCS, National Cancer Institute, Aviano, Pordenone, Italy; ^7^ Department of Medicine, University of Göttingen, Göttingen, Germany; ^8^ Sanct-Josef-Hospital Cloppenburg, Department of Hematology and Oncology, Cloppenburg, Germany; ^9^ Department of Animal Molecular Physiology, Institute of Experimental Biology, Wroclaw University, Wroclaw, Poland; ^10^ Department of Cell Stress Biology, Roswell Park Cancer Institute, Buffalo, NY, USA; ^11^ Dipartimento di Scienze Biomediche e Neuromotorie, Università di Bologna, Bologna, Italy; ^12^ Consiglio Nazionale delle Ricerche, Istituto di Biomedicina e Immunologia Molecolare “Alberto Monroy”, Palermo, Italy

**Keywords:** GSK-3, cancer stem cells, Wnt/beta-catenin, PI3K, Akt, mTOR, Hedgehog, Notch, Targeted Therapy, Therapy Resistance, Mutations, Rapamycin

## Abstract

The serine/threonine kinase glycogen synthase kinase-3 (GSK-3) was initially identified and studied in the regulation of glycogen synthesis. GSK-3 functions in a wide range of cellular processes. Aberrant activity of GSK-3 has been implicated in many human pathologies including: bipolar depression, Alzheimer's disease, Parkinson's disease, cancer, non-insulin-dependent diabetes mellitus (NIDDM) and others. In some cases, suppression of GSK-3 activity by phosphorylation by Akt and other kinases has been associated with cancer progression. In these cases, GSK-3 has tumor suppressor functions. In other cases, GSK-3 has been associated with tumor progression by stabilizing components of the beta-catenin complex. In these situations, GSK-3 has oncogenic properties. While many inhibitors to GSK-3 have been developed, their use remains controversial because of the ambiguous role of GSK-3 in cancer development. In this review, we will focus on the diverse roles that GSK-3 plays in various human cancers, in particular in solid tumors. Recently, GSK-3 has also been implicated in the generation of cancer stem cells in various cell types. We will also discuss how this pivotal kinase interacts with multiple signaling pathways such as: PI3K/PTEN/Akt/mTORC1, Ras/Raf/MEK/ERK, Wnt/beta-catenin, Hedgehog, Notch and others.

## Multiple Roles of GSK-3 in Cellular Physiology and Human Health

GSK-3 is a serine (S) /threonine (T) kinase. GSK-3 was initially isolated and purified from rat skeletal muscle as a kinase that phosphorylated and inactivated glycogen synthase (GS), the last enzyme in glycogen biosynthesis [1,2]. Thus GSK-3 was identified initially to have important roles in metabolism. GSK-3 is believed to be an important regulatory enzyme in many diseases and disorders such as: cancer and aging (cancer stem cells, cellular senescence, control of stem cell pluripotency and differentiation), immune disorders, metabolic disorders (atherosclerosis, diabetes, and heart disease), neurological disorders (Alzheimer's, amyotrophic lateral sclerosis [ALS], bipolar disorder, mood disorders, Parkinson's, and schizophrenia), and other maladies [3-26]. GSK-3 may be a key therapeutic target for these and other diseases [11-26].

GSK-3 has been implicated to play roles in cancers which are resistant to chemo-, radio-, and targeted therapy [7]. Targeting GSK-3 may be a means to overcome the resistance of these cancers to certain chemotherapeutic drugs, radiation and small molecule inhibitors [8-10].

## GSK-3alpha and GSK-3beta

**Figure 1:**
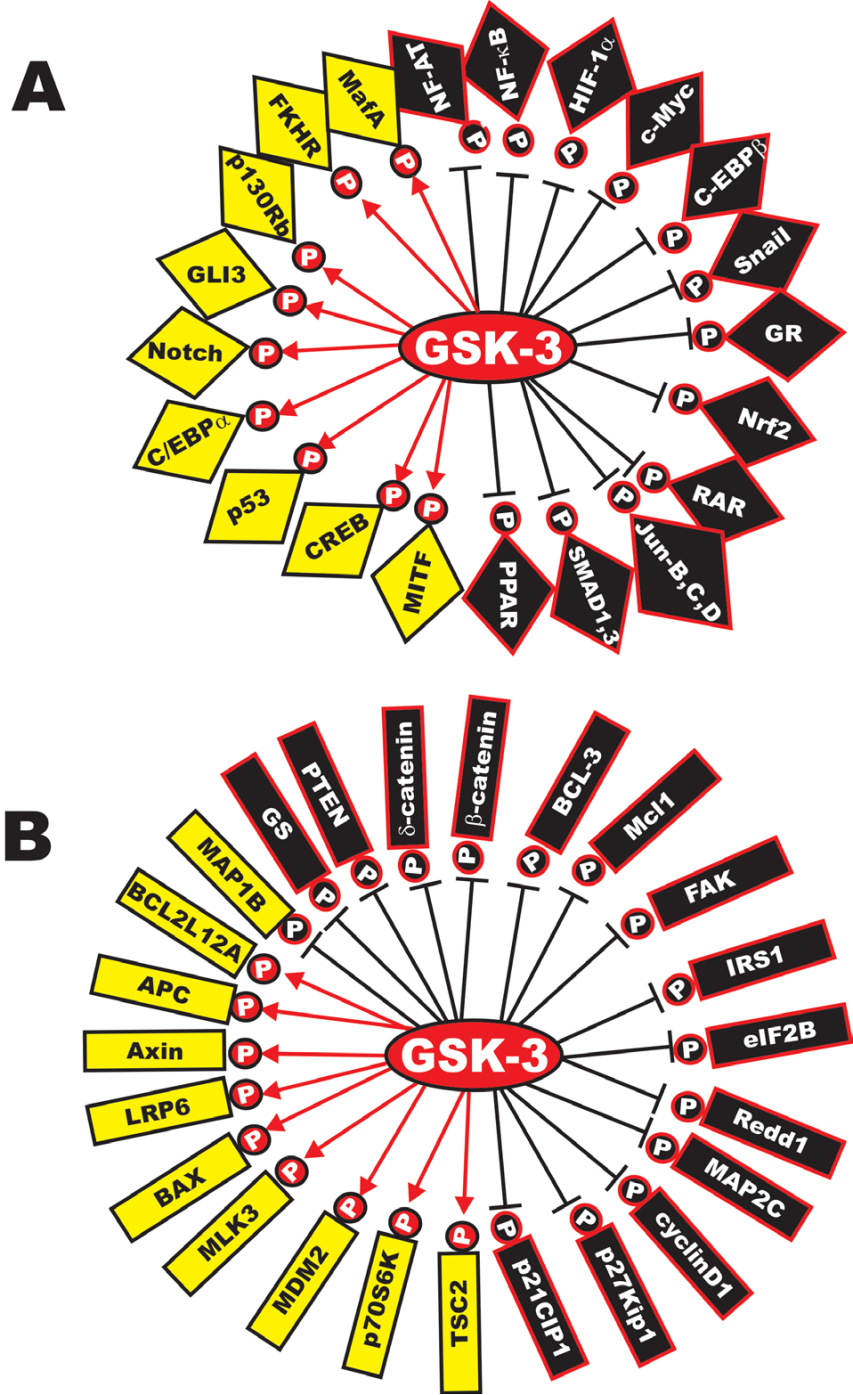
Diversity of GSK-3 Substrates Panel A. Transcription factors phosphorylated by GSK-3. Transcription factors activated by GSK-3 are indicated in yellow diamond type shape with black lettering and P's in red circles. Transcription factors inactivated by GSK-3 are indicated in black diamonds with white lettering and P's in black circles. Panel B. Various proteins phosphorylated and activated by GSK-3 are indicated in yellow rectangles with black lettering and P's in red circles. Various proteins phosphorylated and inhibited by GSK-3 are indicated in black rectangles with white lettering and P's in black circles. In some cases, an individual protein may be activated or inhibited by GSK-3 phosphorylation. This diagram is intended to provide the reader an idea of the diversity of GSK-3 substrates and the roles of GSK-3 in regulating the activity of these substrates.

The GSK-3 gene family consists of two highly conserved kinases GSK-3alpha and GSK-3beta. However, the two molecules have distinct roles and one can not compensate for the loss of the other. In fact, expression of GSK-3alpha did not rescue embryonic lethality in mice in which GSK-3beta gene has been disrupted. *GSK3A* and *GSK3B* encode 51 and 47 kDa proteins respectively [28]. The GSK-3alpha isoform has a glycine-rich extension at its amino terminus. GSK-3alpha and GSK-3beta display 98% sequence identity in their kinase domains but only 36% identity in their carboxyl termini [29]. GSK-3alpha and GSK-3beta are considered active in non-stimulated cells. Both GSK-3-alpha and GSK-3beta exhibit strong preferences for primed substrates; this means they prefer substrates which have already been phosphorylated by other kinases [(e.g., casein kinase-1 (CK1), mitogen activate protein kinases (MAPK) [extracellular regulated kinase (ERK), p^38^^MAPK^, and c-Jun N-terminal kinase (JNK)], 5' adenosine monophosphate-activated protein kinase (AMPK)] and others. The GSK-3 kinases phosphorylate greater than 40 proteins including over 12 transcription factors [30]. Figure 1 presents a diagram indicating some of the substrates of GSK-3.

## Differences between GSK-3alpha and GSK-3beta

GSK-3alpha and GSK-3beta are structurally similar, however, they are not functionally identical and they have some different substrate specificities. These GSK-3beta knock-out mice die around embryonic day 16 due to liver degeneration caused by hepatocyte apoptosis [27]. Furthermore, GSK-3beta activity was essential for TNF-alpha-induced NF-kappaB activation in hepatocytes. In contrast GSK-3alpha knockout mice are viable but exhibited enhanced glucose and insulin sensitivity and reduced fat mass. GSK-3alpha knock-out mice elicited metabolic and neuronal developmental abnormalities [31,32]. GSK-3alpha and GSK-3beta have different substrate preferences in the brain [33] and likely other tissues. Thus, GSK-3 isoforms exhibit tissue-specific physiologically important functions which are may not be overlapping and sometimes may be different. These and other studies indicate that there are rationales for the specific targeting of GSK-3alpha or GSK-3beta in certain diseases.

Most biochemical studies have focused on GSK-3beta; however, some studies have demonstrated roles for GSK-3alpha in drug resistance and cancer stem cells. GSK-3alpha was recently identified as a key target in acute myeloid leukemia (AML) [34]. Thus the generation of isoform specific inhibitors could result in more specific treatments.

## GSK-3 Activity is Controlled by Phosphorylation/Dephosphorylation

**Figure 2:**
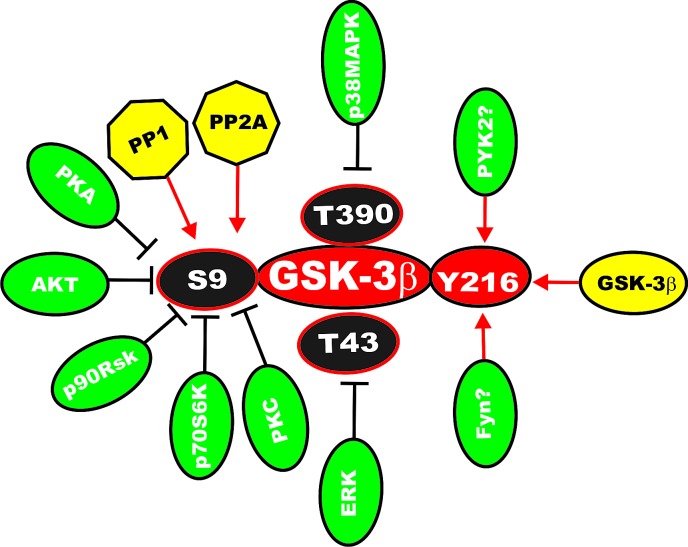
Sites of Phosphorylation of GSK-3beta which Regulate its Activity Kinases which phosphorylate GSK-3beta which result in its inactivation are indicated by yellow ovals with inhibitory black lines. Phosphatases such as PP1 and PP2A have been reported to dephosphorylate S9 which could activate GSK-3beta are indicated in yellow octagons with black arrows. The Y216 site of GSK-3beta has been reported to be phosphorylated by Fyn and PYK2; these are indicated by green ovals with red arrows. Finally, GSK-3beta may autophosphorylate itself at Y216, which would lead to its activation; this is indicated by a yellow oval with a red arrow. This figure is provided to give the reader an idea of the complexity of regulation of GSK-3 by various kinases and phosphatases.

GSK-3alpha and GSK-3beta are expressed ubiquitously and highly conserved. Their activities are regulated by diverse stimuli and signaling pathways. The activity of GSK-3alpha is extinguished by phosphorylation at S21, while GSK-3beta activity is silenced by phosphorylation at S9. These phosphorylation events at S21 and S9 inhibit GSK-3 activity by inducing a pseudosubstrate conformation in the substrate docking motifs of GSK-3alpha and GSK-3beta respectively [28-30]. Phosphorylation of GSK-3beta at S9 leads to its inactivation by proteasomal degradation and has been associated with many pathological conditions, including cancer. Various kinases phosphorylate GSK-beta at S9 including protein kinase A (PKA), protein kinase B (PKB a.k.a Akt), p90 ribosomal S6 kinase (p90^Rsk^), p70 ribosomal S6 kinase (p70S6K) [28-30, 35-39]. A diagram depicting sites of regulation of GSK-3beta is presented in Figure 2.

Insulin signaling results in activation of Akt and subsequent phosphorylation and inactivation of GSK-3beta (S9) and GSK-3alpha (S21) [37]. Epidermal growth factor (EGF), platelet derived growth factor (PDGF) and other growth factors also induce the phosphorylation of GSK-3beta (S9) and GSK-3alpha (S21) by the activated Raf/MEK/ERK/p90^Rsk1^ signaling pathway [39]. Other signaling pathways induce the phosphorylation and inactivation of GSK-3beta and GSK-3alpha by phosphorylation at S9 and S21 respectively [37-42]. Thus GSK-3 activity is often shut off after exposure to mitogenic/growth factors.

Elucidation of the crystal structure of GSK-3 has helped to reveal how the phosphorylation of GSK-3beta and GSK-3alpha at S9 and S21 respectively results in their inhibition [29]. When either S9 or S21 of GSK-3beta or GSK-3alpha respectively, are phosphorylated, a primed pseudosubstrate is created which folds and binds the positively-charged pocket. This folding prevents the phosphorylation of substrates as the catalytic groove is blocked. This is a competitive mechanism of inhibition and if elevated concentrations of the substrates are present, they will out-compete the pseudosubstrate and the substrates will be phosphorylated [43].

Arginine 96 of GSK-3beta is a critical component of the positive pocket which binds primed substrates. Mutation of arginine (R) 96 of GSK-3beta to alanine (A) (R96A) has consequences on the ability of GSK-3beta to phosphorylate primed vs. unprimed substrates. Priming of substrates usually occur N+4 from the site where GSK-3 phosphorylates the substrate [29]. Namely if the substrate is primed, which is a preference of the GSK-3s; they will not be phosphorylated when R96 is mutated to A, whereas if they are not primed they will be phosphorylated. The R96A mutation disrupts the GSK-3beta pocket of positive charge and prevents the phosphorylation of the primed substrates. The R96A mutation prevented the S9-phosphorylated GSK-3beta pseudosubstrate from folding back and resulted in the phosphorylation of unprimed substrates even when GSK-3beta was phosphorylated at S9 [44]. A peptide of eleven amino acids corresponding to the phosphorylated amino terminal of GSK-3beta inhibited competitively the phosphorylation of both primed and unprimed substrates [43]. In contrast, a truncated version of this peptide only inhibited phosphorylation of primed substrates. These results point to different approaches to specifically inhibit the ability of GSK-3beta to phosphorylate different substrates.

Phosphorylation of GSK-3beta at tyrosine (Y) 216 also regulates it activity and may activate GSK-3beta. This may be due to autophosphorylation [45] but it may also be mediated by kinases such as Src. Y279 is the corresponding residue present in GSK-3alpha. Phosphorylation of GSK-3beta at Y216 may be constitutive in resting cells [45,46]. Apoptotic stimuli can increase Y216 phosphorylation of GSK-3beta indicating roles for GSK-3beta in cell death [47-52]. The proline-rich tyrosine kinase 2 (PYK2) may phosphorylate GSK-3alpha and GSK-3beta at Y216 and Y270 respectively [53]. PYK2 controls lysophosphatidic acid-induced activation of GSK-3 which regulates the phosphorylation of microtubule-associated proteins. PYK2-mediated activation of GSK-3 destabilized microtubules during actinomyosin-driven neurite retraction [54,55]. The Fyn tyrosine kinase also phosphorylates GSKs [45,56]. The MAPK family also regulates the activity of GSK-3s. p38MAPK phosphorylates GSK-3beta at S389/T390 [57]. ERK phosphorylation of GSK-3beta at T43 has been proposed to induce a conformational change in GSK-3 which alters its activity [30]. Finally protein phosphatases (*e.g*., PP2A, PP1) have important roles in regulation of GSK-3 activity by removing the phosphate on S9 as well as other regulatory residues [58,59]. In addition, GSK-3s likely have protein phosphatases as substrates (*e.g*., PP1G) [30].

## Biochemical Functions of GSK-3

GSK-3beta represses the expression of certain immediate response genes in quiescent cells [60]. GSK-3 can alter the activity of proteins important in RNA translation such as p70S6K [61,62]. The GSK-3-related yeast protein Mck1 can inhibit the activity of the major mitotic cyclin-Cdk complex Clb2-Cdk1 and regulate cellular division [63]. A diagram illustrating some of the targets of GSK-3 was presented in Figure 1.

Cell cycle arrest at the G_1_ phase occurs upon inhibition of GSK-3 and subsequent activation of p27^Kip-1^ [64]. GSK-3 can also phosphorylate p21^Cip1^ at T57. This phosphorylation event lead to the proteasomal degradation of p21^Cip1^[65]. In contrast, lack of GSK-3-mediated phosphorylation of cyclin D1 at T286 and cyclin E at S380 suppressed their nuclear export and degradation [66-68].

GSK-3 has many direct and indirect effects on cell proliferation. GSK-3 can regulate the activity of transcription factors. Thus it can has important regulatory roles on cellular growth [69]. The growth arrest and DNA damage 45 (*GADD45)* and *GADD153* genes encode tumor suppressors which are checkpoint inhibitors. GSK-3beta can regulate the activity of the tumor suppressor TP53, which can modulate *GADD45* transcription. *GADD45* is induced by DNA damage and its expression is regulated by TP53. The cell cycle is arrested when *GADD45* is induced. c-Myc can also influence the expression of the *GADD43* and *GADD153* genes. GSK-3 can regulate c-Myc activity [70]. c-Myc can also modulate the expression of cell division cycle 25A (*CDC25*) gene which encodes an important cell cycle regulator gene [69].

GSK-3 regulates the activity of other transcription factors including NF-kappaB [69,71,72], Snail [73,74], Notch [75,76], forkhead [77], CAAT-enhancer binding protein (C/EBP) [78]. GSK-3 modifies the activity of other transcription factors frequently implicated in cancer including: activation protein 1 (AP1) [79] and c-Myc [70]. GSK-3beta can phosphorylate NF-kappaB at S468 which promoted its proteasomal degradation [80]. GSK-3beta also interacted with I-kappaBalpha in epithelial cells [81]. Other studies have indicated that GSK-3beta suppressed NF-kappaB activity by preventing the degradation of IkapaBalpha [82]. Thus GSK-3beta can regulate NF-kappaB activity by multiple mechanisms.

GSK-3beta also affected TP53 activity [83,84]. TP53 and miRNA34 can in turn regulate Snail, which is also regulated by GSK-3 [85]. Loss of TP53 activity resulted in decreased miRNA34 levels which bound to the 3'ends of Snail mRNA and suppressed its expression. Hence in the absence of functional TP53, Snail activity was increased and epithelial-mesenchymal transition (EMT) was promoted. GSK-3 regulated this axis. TP53 and miRNA34 are normally suppressors of Wnt/beta catenin signaling [86].

NF-kappaB and GSK-3 regulate the focal adhesion kinase (FAK) [87]. Some extracellular matrix proteins are indirect targets of Snail, c-Myc and NF-kappaB [73,74]. Certain matrix metalloproteinases are transcriptionally regulated by NF-kappaB, C/EBPs and Snail. Furthermore Snail represses E-cadherin transcription [83]. GSK-3 phosphorylates Snail which leads to its nuclear export [74,89]. Phosphorylation of Snail by GSK-3 can result in the cytoplasmic translocation of Snail and lead to increased E-cadherin expression in oral cancer [90]. Stabilization of Snail protein, by increased GSK-3beta phosphorylation, is also observed during EMT in different solid cancer types [91-93]. Snail stabilization and EMT, through enhanced Akt activity and subsequent GSK-3β phosphorylation, is induced by hepatitis B virus X protein (HBx) in HBV-associated hepatocarcinogenesis and in hepatocellular carcinoma (HCC) patients which have amplification and overexpression of the novel oncogene Maelstrom (*MAEL*) [94,95].

The activity of apoptotic and anti-apoptotic family members are also regulated by GSK-3. Bcl-2 expression is inhibited by GSK-3beta phosphorylation of CREB [96]. The myeloid cell leukemia sequence 1 (Mcl-1) anti-apoptotic factor [97-99] is also regulated by GSK-3. However, some studies indicate that GSK-3 activity may not be required for Mcl-1 degradation [100]. GSK-3 stabilizes the expression of certain anti-apoptotic Bcl-2 family members [101-102]. Gsk-3beta can regulate BCL2L12a anti-apoptotic signaling. BCL2L12A is an anti-apoptotic Bcl-2 family member which is expressed at high levels in glioblastoma [103]. When BCL2L12A is phosphorylated at S156 by GSK-3beta, it is stabilized and has increased anti-apoptotic effects with regards to treatment with the drug staurosporin. The effects of BCL2L12A expression on apoptosis were inhibited when the cells were treated with the GSK-3 inhibitor lithium chloride. Interestingly, GSK-3beta and Mcl-1 inactivation have been shown to be part of the mechanism of action of the anesthetic propofol [104].

The expression of the myeloblastosis transcription factor (c-Myb) is also regulated by GSK-3beta. c-Myb is important in the regulation of *BCL2* transcription. Suppression of GSK-3beta inhibited the expression of c-Myb. c-Myc can form a complex with the lymphoid enhancer-binding factor 1 (LEF-1) transcription factor and bind the promoter regions of the *BCL2* and *BIRC5* (survivin) genes which leads to the prevention of apoptosis [105].

Pro-apoptotic Bim is also a target of GSK-3beta. Suppression of GSK-3beta resulted in increased expression of Bim in pancreatic cancer cells, but not in non-transformed pancreatic epithelial cells. This regulation of Bim is thought to occur via JNK-dependent mechanisms which involve Bim transcription [106].

Bcl-2-associated X protein (Bax) can be phosphorylated by GSK-3beta in neuronal cells. Bax phosphorylation by GSK-3beta stimulated the mitochondrial localization of Bax. This localization of Bax had pro-apoptotic consequences [107]. TP53 activity in colorectal cancer cells (CRCs) can be in part regulated by GSK-3beta. These effects of GSK-3beta on TP53 lead to increased levels of Bax [108]. GSK-3 can activate the v-*maf* musculoaponeurotic fibrosarcoma oncogene homolog (MAF) transcription factor which enhanced its transforming activity [109]. GSK-3 also serves to regulate the levels of beta-catenin, a key molecule involved in normal development as well as cancer progression [110-119].

Thursday Inhibtition of GSK-3beta activity was also shown to be critical for association of two metabolic enzymes: hexokinase II [120 PMID: 21430642] and muscle isoform of fructose-1,6-bisphosphatase (FBP2) [121 PMID: 22154964] with mitochondria which results in the protection of the organella against stress stimuli and the promotion of ATP synthesis.

## GSK-3 as a Tumor-Promoter

GSK-3 has various roles in cancer which even after years of study remain complex and controversial. GSK-3 may play positive roles in cell proliferation and its aberrant expression as a tumor promoter. GSK-3 is overexpressed in various tumor types including; colon, liver, ovarian and pancreatic tumors [122-124].

Suppression of GSK-3beta expression inhibited pancreatic cancer growth and angiogenesis [125]. Decreased levels of Bcl-2 and vascular endothelial growth factor (VEGF) were detected in cells which had GSK-3beta knocked-down. GSK-3 inhibitors may be appropriate for treatment of these types of tumors exhibiting GSK-3 overexpression in which GSK-3 is acting as a tumor promoter.

## GSK-3 as a Tumor-Suppressor

GSK-3 also functions as a tumor suppressor. GSK-3 can suppress the Wnt/beta-catenin pathway by phosphorylating beta-catenin which results in the ubiquitin/proteasome-dependent degradation of beta-catenin. Introduction of kinase-inactive (KI), GSK-3beta mutants which presumably functioned as inhibitors of the endogenous wild-type (WT) GSK-3beta protein stimulated Wnt signaling and mammary tumorigenesis [126]. Enforced expression of kinase-dead (KD) GSK-3beta, which presumably functioned as inhibitors of the WT GSK-3beta protein, promoted tumorigenesis of breast and skin tumors [127]. Overexpression of constitutively-active GSK-3beta mutants, in some studies, increased chemosensitivity, cell cycle arrest, and reduced tumorigenicity of breast cancers [128-130]. Pharmacological inhibition of GSK-3 induced EMT and invasion in breast cancer [89]. In these studies suppression of GSK-3 lead to stabilization of beta-catenin which resulted in elevated c-Myc, cyclin-B1 and survivin expression and promoted proliferation and tumorigenesis. There is perhaps a fine balance with GSK-3 whether it functions a tumor suppressor or tumor inducer.

Expression of KI GSK-3beta in murine mammary glands can function as DN mutation and promote breast cancer development by stabilizing beta-catenin protein expression [126]. The GSK-3beta KI construct stabilized beta-catenin expression and increased its nuclear localization and upregulated the expression of beta-catenin target genes such as cyclin D1. The GSK-3beta KI gene was driven by a mouse mammary tumor virus long terminal repeat (LTR) which contains sequences that promoted high level expression of the introduced gene in the mammary tissue. Expression of the GSK-3beta KI mutant resulted in stabilized beta-catenin expression, beta-catenin nuclear localization, activation of a TCF/LEF-dependent reporter construct and increased cyclin-D mRNA and protein levels.

Cyclin D1 was detected at high levels in approximately 50% of breast cancer patients examined. However only 15-20% of these patient samples had amplification of cyclin D1 [131-133]. beta-catenin transactivation has been associated with cyclin D1 overexpression in breast cancer [134]. Aberrant GSK-3beta expression may lead to increased beta-catenin transcription which increases cyclin D1 expression. Cyclin D1 expression is increased in certain breast cancer patients as GSK-3beta regulates cyclin D1 turnover.

Overexpression of the tumor suppressor Axin gene in transgenic mice lead to decreased expression of cyclin D1 and increased apoptosis in mammary epithelia. In addition, impaired lymphoid development was observed in these [135]. CK2alpha overexpression in transgenic mice resulted in mammary tumorigenesis as under these circumstances CK2alpha promoted Wnt signaling [136].

We observed that the GSK-3(KD) mutant induced doxorubicin resistance in MCF-7 breast cancer cells [137]. However, in the same experiments the constitutively-active A9 GSK-3beta also made the cells slightly more doxorubicin resistant than cells transfected with the GSK-3beta (WT) construct. Moreover, the GSK-3beta(KD) construct made the MCF-7 cells more resistant to tamofixen than the cells transfected with the GSK-3beta(WT) construct. However different results were observed with rapamycin which blocks mTORC. MCF-7 cells transfected with the GSK-3beta(KD) construct were hypersensitive to rapamycin while cells transfected with the constitutively-active GSK-3beta (A9) construct were resistant to rapamycin. The cells containing the GSK-3beta(KD) construct were more sensitive to the GSK-3 inhibitor lithium than cells harboring GSK-3beta(WT) construct.

## Mutations in GSK-3 Targets

Since GSK-3 is a kinase with a specific recognition motif present in substrate proteins that it phosphorylates, mutations can occur in certain genes which prevent GSK-3-mediated phosphorylation of the proteins they encode. The mutant proteins may have altered half-lifes as compared to the WT proteins as they may not be subject to proteasomal degradation. For example, mutations changing T58 of c-Myc prevented the ability of GSK-3 to phosphorylate c-Myc and thus suppressed its proteasomal degradation [138-141]. Genetic mutations changing the GSK-3 phosphorylation sites S33, S37, S45 or T41 present in beta-catenin prevented the ability of GSK-3beta to phosphorylate beta-catenin and thus inhibited its proteasomal degradation. Likewise mutations at S45 of beta-catenin prevented CKI from performing the priming phosphorylation, thus GSK-3 was unable to phosphorylate the other residues of beta-catenin. Thus mutations at GSK-3 or CKI phosphorylation sites which prevent the ability of GSK-3 or CKI to phosphorylate target proteins which can under certain circumstances be oncogenic [142-145].

## The PI3K/PTEN/Akt/mTOR Pathway and GSK-3

**Figure 3:**
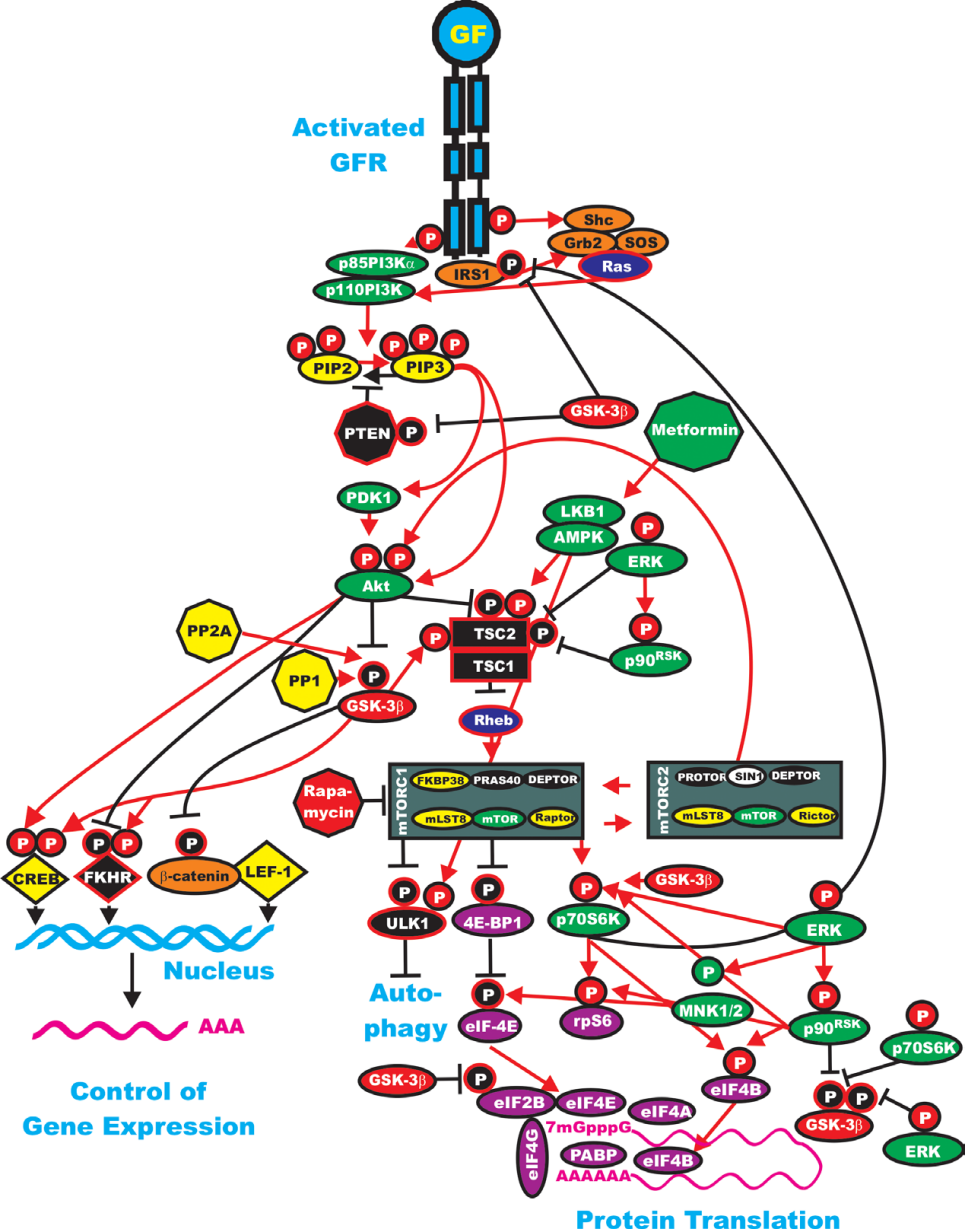
Interactions of GSK-3 with the Ras/PI3K/PTEN/Akt/mTOR and Ras/Raf/MEK/ERK Pathways Some of the regulatory interactions between GSK-3 and Ras/PI3K/PTEN/Akt/mTOR and Ras/Raf/MEK/ERK pathways are indicated. An activated growth factor receptor is indicated in blue. Ras and Rheb are indicated in dark blue ovals. IRS1, Shc, Grb2, Sos and beta-catenin are indicated in orange ovals. Kinases are indicated in green ovals with the exception of GSK-3beta which is indicated in a red oval. The p85 regulatory subunit of PI3K is indicated in a green oval. The phosphatases which inhibit steps in this pathway are indicated in black octagons. The phosphatases PP2A and PP1 which may activate GSK-3 are indicated in yellow octagons. NF1, TSC1 and TSC2 are indicated in black squares. PIP2 and PIP3 are indicated in yellow ovals. The mTORC1 inhibitor is indicated in a red octagon. The AMPK activator Metformin is indicated in a green octagon. mTOR interacting proteins which positively regulate mTOR activity are indicated in yellow ovals. mTOR interacting proteins which negatively regulate mTOR activity are indicated in black ovals. Transcription factors activated by either ERK or Akt phosphorylation are indicated in yellow diamonds. The FKHR transcription factor that is inactivated by Akt phosphorylation is indicated by a black diamond and a white P in a black circle. FKHR is also activated by GSK-3beta phosphorylation which is indicated by a white P in a red circle. beta-catenin is indicated in an orange oval. mRNA initiation factors and proteins associated with the ribosome are indicated in magenta ovals. mTORC1 phosphorylates the unc-51-like kinase 1 (ULK1) which results in the suppression of autophagy. ULK1 is indicated in a black oval. In contrast, AMPK activates both ULK1 and autophagy as well as TSC activity. Proteins involved in the regulation of translation are indicated in purple ovals. Red arrows indicate activating events in pathways. Black arrows indicate inactivating events in pathway. Activating phosphorylation events are depicted in red circles with Ps with a black outlined circle. Inactivating phosphorylation events are depicted in black circles with Ps with a red outlined circle. This figure is provided to give the reader an idea of the complex interactions of GSK-3 with various signaling molecules in the Ras/PI3K/PTEN/Akt/mTOR and Ras/Raf/MEK/ERK pathways which are key in regulating cellular proliferation survival and often become dysregulated in cancer.

The PI3K/PTEN/Akt/mTOR pathway and GSK-3 can cross-regulate each other at various points. Some of the interactions between GSK-3 and the PI3K/PTEN/Akt/mTOR pathway are presented in Figure 3. As stated previously, GSK-3beta is negatively by Akt when it is phosphorylated on S9. GSK-3beta is then inactive.

**Figure 4:**
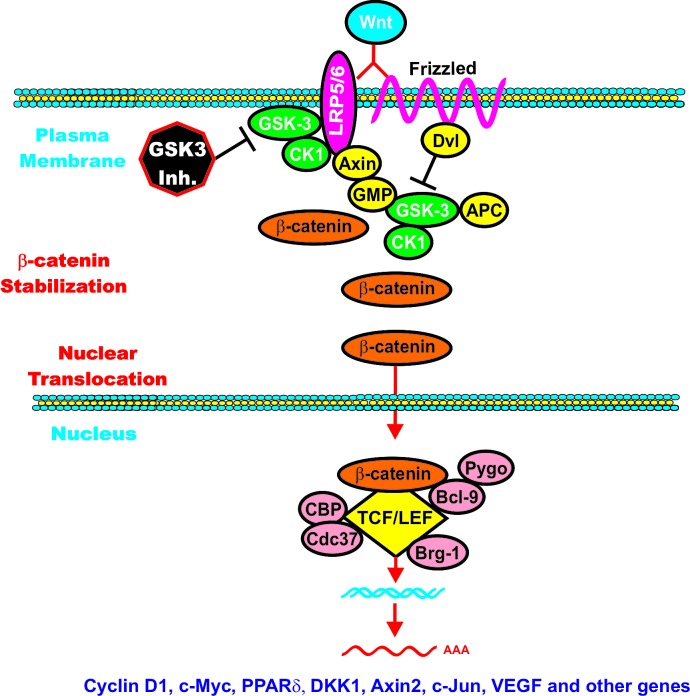
Wnt/beta-catenin Induced Gene Expression is Modulated by GSK-3 When Wnt is present beta-catenin is stabilized and can induce gene transcription. Wnt binds its coreceptors Frizzled (Fz) and LRP5/LRP6. The Fz transmembrane receptor is indicated by a squiggly line to indicate its spanning the membrane seven times. The LRP5 and LRP6 transmembrane receptors are indicated by a red oval. Various molecules which interact with the receptors and GSK-3 and CK1 are indicated in yellow ovals. In the presence of Wnt, beta-catenin is stabilized and induces gene expression by complexing with various transcription factors such as TCF/LET, which are indicated by yellow diamonds. Various proteins which interact with the transcription factor complexes are indicated in pink circles.

The PI3K/PTEN/mTOR pathway is also involved in cancer initiation, metastasis, drug resistance, and sensitivity to therapy [146-152]. PI3K p110 catalytic subunit (*PIK3CA*) mutations may be driver mutations in certain cancers responsible for metastasis [153]. Novel PI3K-alpha inhibitors have been isolated and they inhibit metastasis [154]. Most PI3K inhibitors are cytostatic rather than cytotoxic and it has been questioned whether treatment with a single PI3K inhibitor will be effective [155].

The PI3K/PTEN/Akt/mTORC1 pathway is important in c-Myc expression in Burkitt's lymphomagenesis in germinal center B cells [156]. The PI3K/PTEN/Akt/mTORC1 pathway is also an emerging target for mantle cell lymphoma as this cascade is upregulated in this cancer [157]. Disruption of PTEN and TP53 activity in the thyroid has recently been shown to result in murine models of anaplastic thyroid carcinomas. This model could be important for the development of approaches to target human thyroid carcinomas [158]. The PI3K/PTEN/Akt/mTORC1 pathway is a key pathway involved in many cancers including gliomas often due to aberrant *PTEN* expression. Recently it has been shown that reduction of *PIK3CA* or PI3K p85 regulatory subunit (*PIK3RA*) cell proliferation impedes proliferation, migration, and invasion in glioblastoma multiforme cells [159]. In contrast to *PIK3CA*, *PIK3CB* (PI3K p110-beta catalytic subunit) is oncogenic in its WT configuration when it is overexpressed in certain conditions. *PIK3CB* can act like an oncogenic mutant of *PIK3CA* [160].

*PIK3CD* (PI3K-delta p110 catalytic subunit) has been shown to have roles in BCR-ABL-mediated chronic lymphocytic leukemia (CLL) by turning off breakpoint cluster region (BCR) signaling [162]. Elevated PI3K signaling has been detected in certain breast cancer subtypes and is believed to drive their abnormal proliferation [162]. Novel lipid phosphatases have been shown to be important in regulation of this pathway. Inositol polyphosphate 4-phosphatase type II (*INPP4B*), is a tumor suppressor gene implicated in many cancers including: breast, ovarian and prostate cancers [163].

## mTORC1 and mTORC2 Complexes

The S/T mTOR kinase is a critical component of two complexes, mTORC1 and mTORC2. In the mTORC1 complex, mTOR complexes with Raptor (Regulatory associated protein of mTOR) adaptor protein, DEP domain containing mTOR-interacting protein (DEPTOR) and mLST8, a member of the Lethal-with-Sec-Thirteen gene family, first identified in yeast, FK506 Binding Protein 38 (FKBP38) and proline-rich Akt substrate 40 kDa protein (PRAS40) [150,164,165]. *DEPTOR* may be a tumor suppressor gene as decreased expression of DEPTOR results in increased mTORC1 activity [166]. mTORC2 can function as the elusive PDK-2 which phosphorylates Akt-1 on S473 in response to growth factor stimulation [167].

## LKB1/AMPK Network Interactions with GSK-3 and PI3K/Akt/mTORC1 Pathway

The LKB1/AMP activated protein kinase (LMPK) pathway is a key pathway initially investigated in metabolic diseases such as diabetes. However, the LMPK pathway is now recognized to play important roles in cancer and other diseases [165]. The LKB1/AMPK pathway can be stimulated by the commonly prescribed drugs, such as N,N-dimethylimidodicarbonimidic diamide (metformin) and 5-aminoimidazole-4-carboxamide 1-β-D-ribofuranoside (AICAR). AICAR is used to prevent cardiac ischemic injury and is also used in diabetes care. Activation of LKB1/AMPK in various tumor types results in cell cycle arrest, caspase-dependent apoptosis or autophagy in various tumors. Metformin inhibits mTORC1-controlled protein translation. Many of the targeted proteins are important in the growth of malignant cells. The translation of these proteins is not as well inhibited with allosteric mTORC1 inhibitors, such as rapamycin and its derivatives. Recently it was shown that metformin also targets the cancer initiating cells (CICs), the critical target for cancer eradication. Thus the LKB1/AMPK pathway is critically involved in regulating diseases such as diabetes as well as the proliferation and survival of malignant cells. Drugs targeting and activating LKB1/AMPK maybe a novel, less toxic and cost effective treatment option for certain malignancies [168,169].

The ability of metformin to suppress the proliferation of T-acute lymphoblastic leukemias (T-ALL) cell lines and primary T-ALL patient samples displaying mTORC1 activation was examined. Metformin induced autophagy and apoptosis and inhibited proliferation. The effects of metformin on control CD4+ T-lymphocytes were examined. Metformin was much less toxic against CD4+ T-lymphocytes from healthy donors than against either the T-ALL cell lines or primary patient cells. These studies documented the dephosphorylation of downstream targets of mTORC1. In addition, inhibition of mRNA translation in metformin-treated T-ALL cells was observed. In contrast, a similar inhibition of translation was not observed after rapamycin treatment. Importantly in primary patient samples, metformin targeted the side population of T-ALL cell lines as well as a putative leukemia initiating cell (LIC) subpopulation (CD34+, CD7-, CD4-). Metformin exhibited anti-leukemic activity, which supports further development of LKB1/AMPK pathway activators as potential clinical candidates for T-ALL therapy [170]. AMPK is also important in BCR-ABL-induced chronic myeloid leukemia (CML) and AMPK pathway activators may prove useful as combination drug therapy (with BCR-ABL inhibitors) for this disease [171]. AMPK can also be activated by rapamycin [172].

*LKB1* is an important tumor suppressor and gatekeeper mutations of *LKB1* cause the rare Peutz-Jeghers Syndrome (PJS) which is a cancer-prone syndrome [173]. LKB1 is a critical regulator of cell polarity and energy/metabolism control and exerts it vast effects via diverse effectors [174,175]. AMPK is considered a metabolic gatekeeper important in many diseases including diabetes, cancer and neurologic disorders. Inhibiting mTORC1 activity by drugs such as metformin and other drugs (including rapamycin) may not only aid in the treatment of diabetics, but also improve cancer therapies and increase longevity [176-179].

The plant natural product berberine has recently been shown to inhibit the growth of drug-resistant breast cancer cells whereas the parental drug sensitive line was not as growth inhibited. Berberine may also target LKB1/AMPK. Cells overexpressing neutrophil gelatinase-associated lipocalin (NGAL) were more sensitive to berberine than parental cells which did not over express NGAL [180].

## Regulation of mTORC by GSK-3, TSC1/TSC2 and Other Molecules

The activity of the mTORC1 complex is regulated by many different molecules. Akt can suppress tuberous sclerosis 2 (TSC2 or tuberin) function by phosphorylation [181]. TSC2 is a GTPase activating protein (GAP). TSC2 functions in association with TSC1 (hamartin) to regulate mTORC1 activity. TSC1/TSC2 inactivate the small G protein Ras homolog enriched in brain (Rheb) [182]. TSC2 phosphorylation by Akt represses GAP activity of the TSC1/TSC2 complex, allowing Rheb to accumulate in a GTP-bound state. Rheb-GTP then activates the protein kinase activity of mTOR present in the mTORC1 complex.

GSK-3 plays key roles in this regulatory circuit. The TSC1/TSC2 complex can negatively-regulate proliferation through mTORC1 inhibition and GSK-3beta activation. GSK-3 can also interact with TSC1/TSC2 by phosphorylating TSC2 and activating it [183]. This regulatory loop would result in suppression of mTORC1 activity.

Akt also phosphorylates the proline-rich Akt substrate of 40 kDa (PRAS40), another inhibitor of mTORC1. When PRAS40 is phosphorylated by Akt, it can not suppress mTORC1 signalling [154,154,165,169,183-185]. Thus, this is another mechanism by which Akt activates mTORC1. To complicate matters, PRAS40 is a substrate of mTORC1 itself, and mTORC1-mediated phosphorylation of PRAS40 prevents inhibition of additional mTORC1 signaling. The Ras/Raf/MEK/ERK signaling pathway also regulates mTORC1. TSC2 is phosphorylated by both ERK and p90^Rsk-1^. When TSC2 is phosphorylated by either ERK or p90^Rsk-1^, its inhibitory function is suppressed [183-185]. Moreover, inhibition of mTORC1 can result in ERK1/2 activation, through p70S6K/PI3K/Ras/Raf/MEK [186].

The mechanism(s) by which Rheb-GTP activates mTORC1 are not fully elucidated. However for Rheb to activate mTORC1, Rheb must be farnesylated and farnesylation can be suppressed by farnesyl transferase (FT) inhibitors. Rheb-GTP may relieve the inhibitory function of FK506-binding protein 8 (FKBP38) on mTOR, thus leading to mTORC1 activation [182].

mTORC1 also inhibits Akt via a negative feedback loop which involves p70S6K and potentially other molecules [187]. p70S6K can exert negative effects on insulin receptor substrate-1 (IRS-1) [154,154,165,169,183-185]. p70S6K can phosphorylate IRS-1 on S312 and/or S636/S639. This results in the targeting of IRS-1 to the proteasome where it is degraded. Hence PI3K/Akt signaling downstream of IRS-1 is downregulated when p70S6K is active. Rapamycin blocks mTORC1 and p70S6K activation, thus this loop is broken upon rapamycin was added and Akt is activated.

GSK-3 can regulate this pathway by phosphorylation of S371 on p70S6K [61,62,65,186,187]. GSK-3 interacts with mTORC1 to regulate p70S6K activity [61,62]. GSK-3beta can have negative effects on p70S6K activity [188]. GSK-3 can phosphorylate TSC2 which results in inhibition of mTORC1 and prevents the phosphorylation of p70S6K at T389 [189,190]. Little is known about the interactions of GSK-3 and mTORC2 in oncogenesis. mTORC2 stabilizes cyclin D1 through prevention of GSK3- dependent and FBX4-mediated cyclin D1 degradation [190] Thursday

## Involvement of GSK-3 in Hematopoietic Stem Cell (HSC) Homeostasis

To maintain a balanced number of self-renewing cells or to generate the required lineage-differentiated progeny cells necessary to fulfill the loss of mature blood cells, normal HSCs exit quiescence under the appropriate circumstances. GSK-3 plays key roles in HSC homeostasis by regulating the quiescent HSC pool [91]. Depletion of functional GSK-3 activity in bone marrow (BM) transiently expanded HSC in a beta-catenin-dependent fashion, indicating roles for GSK-3 and Wnt/beta-catenin signaling in homeostasis. Depletion of GSK-3 activity in the HSC pool resulted in their depletion by activation of mTORC1. Loss of the HSCs in the GSK-3-deficient BM could be suppressed by treatment with mTORC1 inhibitors. GSK-3 is a master regulatory switch which controls both Wnt/beta-catenin and mTORC1 signaling which in turn regulates HSC self renewal and lineage commitment respectively. This important study has suggested a potential therapeutic approach to expand HSCs *in vivo* by using already approved drugs for treatment of other diseases (rapamycin/rapalogs to inhibit mTORC1 and lithium to inhibit GSK-3).

As stated previously, GSK-3 phosphorylates TSC2 which in turn inhibits Rheb and mTORC1 activity. The PTEN phosphatase is upstream of GSK-3. PTEN negatively regulates Akt activity by de-phosphorylating phosphatidylinositol (3,4,5)-trisphosphate (PIP3) which Akt requires for membrane localization. *PTEN* and *TSC1* knock-out mice have similar hematopoietic phenotypes. Increases in HSC were transiently observed which were followed by HSC depetion and lineage commitment [192,193]. The authors suggested that there were additional tumor suppressors which were induced in *PTEN-*mutant mice indicating the complicated interacting genetic factors between *PTEN* loss and mTOR activation [194]. Knock-down of GSK-3alpha and GSK-3beta induced a similar HSC phenotype as observed in *PTEN−/−* and *TSC-1−/−* mice as the HSCs initially increased and then they were progressively depleted [195]. Thus GSK-3 plays critical roles in HSC homeostasis and regulating the decision between self-renewal and differentiation. These pathways often have competing functions in HSC self renewal and differentiation. Wnt signaling can induce mTORC1 activity by preventing the inhibitory effects that GSK-3 has on mTORC activity. Thus there is a division of the canonical Wnt pathway with Wnt or GSK-3 inducing opposing signal pathways. Wnt and GSK-3 can activate potentially opposing processes (HSC renewal vs. differentiation). Other factors and molecules may also be discovered that may modify the balance and modify HSC homeostasis.

Nutrient-sensing pathways interact to regulate HSC homeostasis. HSCs reside in the BM in a nutrient and oxygen-deprived niche. Both the mTORC1 and LKB1 pathways are important nutrient-sensing pathways. Dysregulation of these pathways alters HSC homeostasis. Increased mTORC1 activity or decreased LKB1 activity [193,196-199] stimulates the proliferation of committed progenitors and decreases HSCs. Low nutrient availability is a critical component which controls HSC homeostasis.

Suppression of this pathway by rapamycin combined with activation of Wnt/beta-catenin pathway by GSK-3 inhibitors permitted *ex vivo* maintenance of human and mouse HSCs in the absence of exogenous cytokines. These studies demonstrated that two drugs (GSK inhibitor CHIR99021 and the mTOR blocker rapamycin) increased the number of long-term HSC *in vivo* [200]. Thus GSK-3 plays roles in HSC homeostasis as a suppressor of Wnt/beta catenin self-renewal pathway and also as a suppressor of the mTORC1 pathway which would normally promote lineage commitment and stem-cell depletion. Inhibition of GSK-3 activated self renewal while suppression of mTORC1 prevented HSC depetion allowing the generation of long term HSC (but not the proportion) *in vivo*. This approach may also prove useful for both hematopoietic recovery and immune reconstitution in umbilical cord blood transplantation.

The promyelocytic leukemia (PML)–peroxisome proliferator-activated receptor-delta (PPAR-delta)–fatty-acid oxidation (FAO) pathway is another pathway which is important in HSC maintenance [201]. GSK-3 regulates this pathway. PPARs are regulated by GSK-3 phosphorylation [202]. Loss of HSC maintenance can occur after suppression of PPAR-delta or inhibition of mitochondria FAO. Improved HSC maintenance was observed after treatment with PPAR-delta agonists. PML regulates PPAR signaling and FAO which have important roles in HSC maintenance [201]. The asymmetric division of HSCs is controlled by this pathway. Thus the PPAR-FAO pathway is a metabolic regulatory switch for the control of HSC fate. PPARs are important transcription factors that regulate nutrient sensing and metabolic pathways including FAO and fatty acid transport. These studies have important therapeutic implications as small molecule PPAR activators and inhibitors have been developed and evaluated for the treatment of obesity and metabolic disorders [203,204].

The *PML* gene is a tumor suppressor gene originally cloned from the break point of the t(15;17) chromosomal translocation present in PML. PML has an important role in regulating the maintenance of HSCs and LIC quiescence. Suppressing the activity of PML eliminated certain LICs [205]. *PML*-deficient CML-LICs became exhausted over time and lost their ability to generate CML in transplanted mice. PML also acted as a negative regulator of mTORC1 in a mouse CML model [206-207]. Repressing or balancing mTORC1 activity is important in HSC maintenance. Elevated mTORC1 activity adversely affected LIC maintenance while the mTORC1 blocker rapamycin prevented the exhaustion of *Pml^−/−^* LICs. PML is targeted for degradation by arsenic trioxide. Arsenic trioxide treatment of murine CML models decreased PML expression and prevented maintenance of CML-LIC quiescence.

GSK-3 inhibition permitted mouse embryonic stem cells (ESC) to maintain pluripotency. ESCs prepared from mice lacking GSK-3alpha and GSK-3beta retained some markers of pluripotency even when they were cultured under differentiation-inducing conditions [208-209].

GSK-3 and mTORC1 may also regulate T cell memory. Importantly inhibition of GSK-3, mTORC1 or both may augment the number and competence of CD8+ memory T cells [210-212].

## Involvement of GSK-3 and Wnt/beta-catenin Signaling

**Figure 5:**
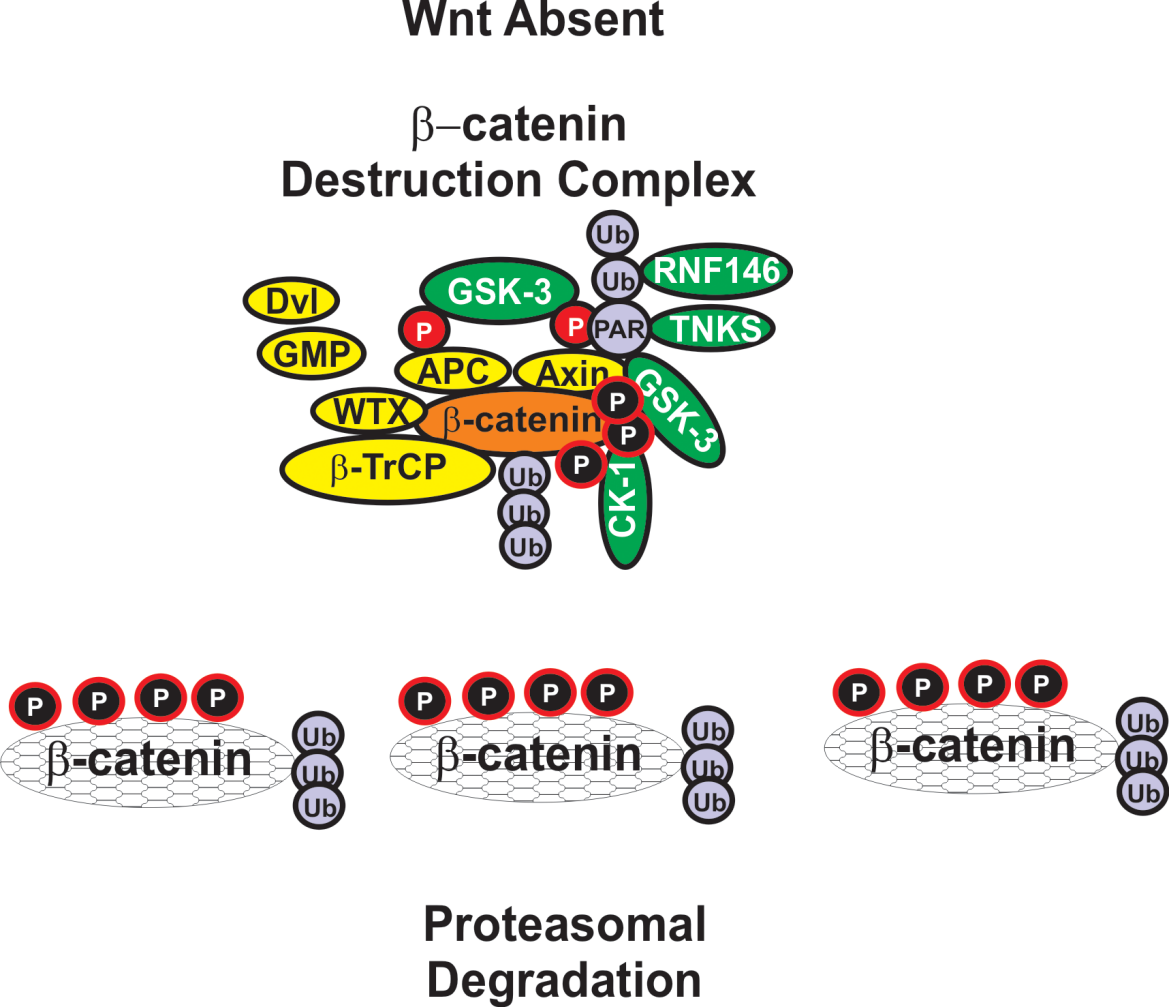
Formation of beta-catenin Destruction Complex When Wnt is absent, beta-catenin is targeted for proteasomal degradation. GMP, indicated in a yellow oval, is displaced from GSK-3 and GSK-3 is able to phosphorylate beta-catenin which results in its ubiquitination by the beta-TrCP complex which is indicated in a yellow oval. Ubiquitination is indicated by Ub in purple circles. Axin is also poly-ADP-ribosylated (PAR) by tankyrase (TNKS) in a green oval with white letters. PAR is indicated in a purple circle which subsequently leads to the proteasomal degradation of Axin. Axin is also ubiquitinated by RNF146 RING-type ubiquitin E3 ligase (RNF146) is indicated by a green ovals with white letters.

Essentially there are two Wnt signaling pathways, a canonical Wnt signaling pathway which is beta-catenin-dependent and a non-canonical Wnt signaling pathway which is beta-catenin-independent. Beta-catenin can play multiple roles in cell physiology. Different pools of beta-catenin likely exist at various subcellular locations. For example one pool of beta-catenin is associated with cadherins at the cell-cell junctions while another pool of beta-catenin is “free” and localized in the cytosol and nucleus where it can play important roles in regulating gene expression. “Free” beta-catenin exists at a low level and is regulated by rapid turn-over. GSK-3 is critical in regulating the turnover of beta-catenin. An overview of the interactions between GSK-3 and the Wnt/beta-catenin pathway is presented in Figure 5.

The Wnt gene family is comprised of approximately nineteen genes in mammals. Wnt genes are found in multicellular animals but not in single-cell organisms suggesting that the Wnt genes played essential roles in the evolution of multicellular organisms [213]. When Wnt binds it's receptor, a conformation change of the receptor occurs resulting in the phosphorylation of target proteins. Wnt binds a member of the Frizzled (Fz) family which is comprised of approximately 10 family members. Fz proteins are membrane bound receptors which are G-protein coupled receptors (GPCRs). The low density lipoprotein receptor (LDR) related proteins 5/6 (LRP5 or LRP6) are transmembrane proteins which are co-receptors for Wnt. Additional proteins can interact with Wnt to transduce various signals, these include receptor tyrosine kinases (RTKs), such as receptor-tyrosine-kinase-like orphan receptor 1 (ROR1) and ROR2 as well as the RTK-like proteins [214].

The GSK-3 *Drosophila* homologue is zeste-white 3, a negative regulator of wingless (Wg) signaling which is critical for normal development. Wnt is the vertebrate homologue of Wg and the Wnts are responsive for embryonic patterning as well as many other processes and their dysregulation can result in cancer [215]. Wnt signaling is complex as at least 19 different Wnts exist in mammals and depending on the cellular context, different Wnts can induce or suppress critical aspects of proliferation. Activation of the canonical Wnt/beta-catenin pathway results in the induction of T cell factor/lymphoid enhancer factor-1 (TCF/LEF)-responsive genes. beta-catenin serves as a transactivator which binds to TCF/LEFs which are bound to specific gene targets in the DNA.

GSK-3 normally inhibits this pathway as in the absence of Wnt ligands; GSK-3 phosphorylates the amino terminals domain of beta-catenin which targets it for ubiquitination and proteasomal degradation. However, when Wnt ligands are present, GSK-3 is inactivated. The disheveled (Dvl) phosphoprotein is required to transduce a signal from Wnt that results in the inactivation of GSK-3beta [216]. As a result, beta-catenin is dephosphorylated and accumulates in the cytoplasm and translocates to the nucleus where it associates with TCF/LEFs to become a transcriptional co-activator [29].

## Regulation of beta-Catenin Activity by GSK-3

In the absence of stimulation, CK1 phosphorylates beta-catenin at S45 which primes beta-catenin for subsequent phosphorylation by GSK-3 at S41, S37 and S33 [29,143,217-221]. These phosphorylation events target beta-catenin for ubiquitination and proteasomal degradation [29]. A blow up of the Wnt/beta-catenin and beta catenin destruction complex is presented in Figure 5.

When Wnt ligand is present, it binds Fz, which signals through Dvl to suppress beta-catenin phosphorylation. beta-catenin is then able to complex with LEF/TCF and induces the transcription of genes. When beta-catenin is mutated at certain nucleotides it can act as an oncogene, as the beta-catenin proteins can not be phosphorylated by GSK-3 or CKI (depending on the residue). Other regulatory proteins are also involved in controlling the activity of the Wnt/beta-catenin complex. In many cases, they may be either oncogenes or tumor suppressor genes. GSK-3-binding protein (GBP), also known as, frequently rearranged in advanced T-cell lymphomas (FRAT), may regulate the binding of GSK-3 to Axin. Diversin (Div) is an ankyrin rich protein that interacts with both Dvl and Axin to promote Wnt signaling. The beta-catenin destruction complex consists of: beta-catenin, axin/conductin, adenomatous polyposis coli (APC), CK1, GSK-3 and GBP. APC is a tumor-suppressor gene and an essential component of the beta-catenin complex which controls cytoplasmic beta-catenin levels. Mutations at APC are detected in certain cancers such as colo-rectal cancer (CRC). Mutations at APC occur early in CRC and result in increased beta-catenin levels which lead to the expression of Wnt/beta-catenin responsive genes. The Axin and related conductin (Axil) proteins contain multiple protein:protein interaction domains. The Axin/Axil proteins interact with APC and form key components of the beta-catenin destruction complex. Axin and APC are both phosphorylated by GSK-3. Phosphorylation of Axin by GSK-3 increases it stability as well as its ability to bind to beta-catenin [222-225]. CKI is a priming kinase by phosphorylating Axin, Dvl and APC. Once these proteins are phosphorylated by CK1, GSK-3 then phosphorylates them. The Div protein can recruit CKI to the beta-catenin destruction complex [226].

GBP plays key important roles in the interactions between GSK-3 and other proteins. The binding sites for Axin and GBP on GSK-3 are overlapping. When GSK-3 is bound to GBP, GSK-3 cannot bind Axin and thus GSK-3 does not phosphorylate beta-catenin [227,228]. GBP can regulate the nuclear export of GSK-3 [229] and control the accessibility of nuclear and cytoplasmic substrates to GSK-3.

**Figure 6:**
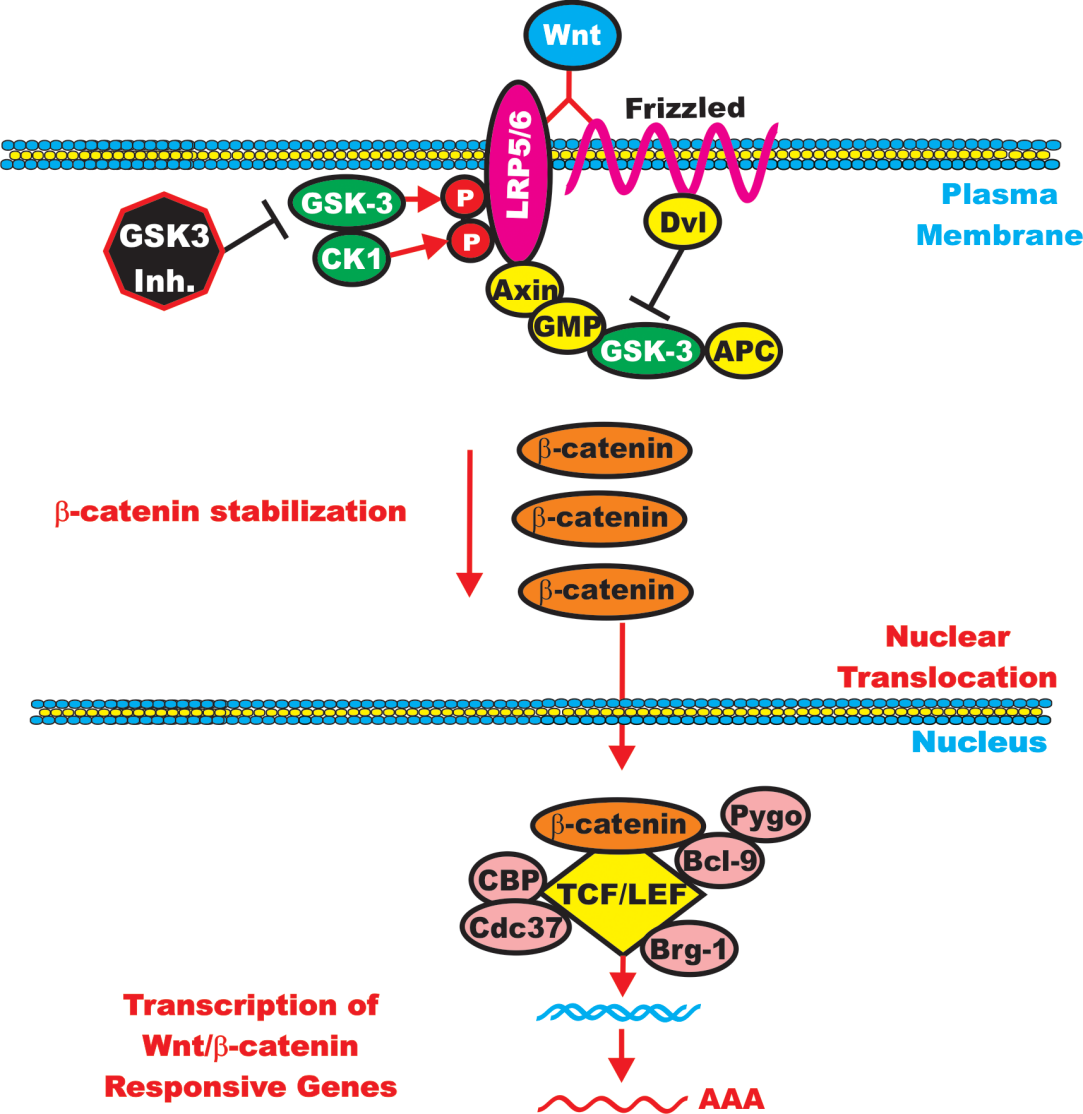
GSK-3 can Enhance Wnt/beta-catenin signaling GSK-3 and CK-1 can phosphorylate and stabilize LRP5/6 which promotes beta-catenin signaling. GSK-3 inhibitors could inhibit beta-catenin–mediated gene expression. GSK-3 inhibitors are indicated by a black octagon with white lettering.

Dvl attaches to the cytoplasmic portion of Fz. Wnt/Fz then associates with the Wnt-coreceptor LRP5 or LRP6. CKI phosphorylates and primes LRP6 on T1479. GSK-3beta then plays a positive role in Wnt signaling by phosphorylating LRP6 on S1490. These events are required for Axin to bind to the complex which results in beta-catenin stabilization. beta-catenin can then enter the nucleus and effect gene transcription [230-235]. Figure 6 presents a diagram of the positive effects which CKI and GSK-3 exert on Wnt/beta-catenin signaling. Targeting GSK-3 may be effective in suppressing the growth of certain cancers.

Axin attaches to the cytoplasmic portion of LRP6. This complex sequesters beta-catenin away from the beta-catenin destruction complex and suppresses the ability of GSK-3beta to phosphorylate beta-catenin. If Wnt signaling is turned on, GSK-3-mediated phosphorylation of S33, S37 and T41 on beta-catenin is suppressed and beta-catenin is stabilized.

GSK-3 has two roles in regulation of Wnt/beta-catenin signaling. Phosphorylation of LRP5/6 stabilizes Axin while phosphorylation of beta-catenin targets it for proteasomal degradation. As stated previously, there may be multiple pools of GSK-3 in cells; one linked with Axin and is resistant to phosphorylation by Akt. Another pool of GSK-3 is regulated by Akt and when it is phosphorylated by Akt it is destabilized and degraded [230]. Introduction of constructs encoding activated Akt did not activate beta-catenin signaling in the examined model system in terms of changes in levels of LEF1-dependent transcription [231]. Wnt may induce GSK-3 and Axin translocation to the plasma membrane. These translocations may be dependent upon interactions with Fz and Dvl [232-234]. Axin is required for GSK-3 to phosphorylate LRP5/6. Wnt signaling induces the association of CKI with LRP5/6. This association may also be involved in Wnt-induced Axin recruitment [290].

CKI phosphorylates beta-catenin on S45 when Wnt signaling is extinguished. GSK-3beta can then phosphorylate beta-catenin on S33, S37 and T41. The Axin/GSK-3/CK1/APC complex induces the degradation of beta-catenin. This occurs via ubiquitination by the SKP1/cullin1-F-box E3 ligase (beta-TrCP) complex in the 26S proteasome. The tumor suppressor APC interacts with both GSK-3 and beta-catenin. Axin serves as the scaffold of the destruction complex. APC binds directly to Axin. Axin is phosphorylated by CKI and then GSK-3 [291]. The affinity of beta-TrCP for beta-catenin is normally low. The Wilms tumor suppressor (WTX) protein increases the affinity of beta-TrCP for beta-catenin [237]. A complex is formed consisting of and APC, Axin, beta-catenin, beta-TrCP and WTX, which promotes beta-catenin ubiquitination and subsequent proteasomal degradation.

Axin, Apc and WTX are tumor-suppressor proteins. APC increases the activity of GSK-3 [238]. GSK-3 phosphorylates conserved serines present in the PPPSP motif found in many Wnt signaling components such as beta-catenin, Axin, and APC.

## GSK-3 and beta-Catenin Signaling and Hepatocarcinogenesis

The dysregulation of GSK-3beta phosphorylation and inhibition of GSK-3beta activity has been shown to be involved in hepatocarcinogenesis [239]. In a recent study performed on 80 HCC patients, the expression of GSK-3beta protein in HCC tissues was found to be significantly lower than that in normal liver tissues and pericancerous tissues [240]. In addition, low expression of GSK-3beta was correlated significantly with advanced clinicopathological characteristics and poor prognosis of HCC patients. GSK-3beta expression was also correlated with vascular invasion, histological grade, TNM classification and therefore could be involved in process of HCC metastasis [241].

In a different study, it was reported that some HCC cells expressed high basal levels of S9-phosphorylated GSK-3beta [242]. As a result of the sustained phosphorylation of GSK-3beta, beta-catenin was stabilized and contributed to hepatic transformation.

Infection with either HBV or HCV are well known risk factors for HCC. HBx is one of the four proteins encoded by the HBV genome. Several studies have reported its involvement in liver carcinogenesis. Expression of HBx in hepatocytes activates the Ras/Raf/MEK/ERK signaling cascade. Interestingly, cross-talk between the Ras/Raf/MEK/ERK and Wnt/beta-catenin signaling has been observed in hepatoma cells [243]. HBx activates ERK and as consequence it associates and primes GSK-3beta for its inactivation. In particular, activated ERK phosphorylated GSK-3beta at T43, which primers GSK-3beta for its subsequent phosphorylation at S9 by p90^Rsk^, resulting in inactivation of GSK-3beta. In turn, inactivation of GSK-3beta promoted beta-catenin stabilization and signaling [243]. In other scenarios, ERK can collaborate with GSK-3 in the phosphorylation of paxillin and regulate cytoskeletal rearrangement [244].

Among the HCV encoded proteins, the core protein has been reported to contribute to HCC carcinogenesis. Interestingly, HCV core protein increases and stabilizes beta-catenin levels in hepatoma cells through inactivation of GSK-3beta by phosphorylation at S9. This results in stabilization of beta-catenin and enhancement of canonical Wnt/beta-catenin signaling activity. Downstream beta-catenin target genes, such as *c-Myc*, *cyclin D1*, WNT1-inducible-signaling pathway protein 2 (*WISP2*) and connective tissue growth factor (*CTGF*) are expressed at higher levels [245].

GSK-3beta/beta-catenin signaling plays roles in the Sirtuin 2 (SIRT2)-mediated transformation in HCC [246]. SIRT2 is a member of sirtuin family, which is upregulated in about 50% of HCC patients. Increased SIRT2 expression is correlated with shorter overall survival [246]. The sirtuins have been associated with oxidative stress and aging [247]. The authors demonstrated that SIRT2 regulated activation of Akt by its deacetylation [246]. Subsequently Akt phosphorylated and inactivated GSK-3beta. The beta-catenin-signaling cascade is enhanced and promoted EMT and cell migration of HCC cells. Together these results suggested that GSK-3beta is probably a tumor suppressor gene in HCC, and the loss of GSK-3beta expression and/or activity may contribute to development of HCC.

However, the roles of GSK-3beta in HCC remain controversial and several studies indicate that GSK-3beta is a potential therapeutic target for a broad spectrum of cancer types [248-253] including HCC [254.^255^].

Inhibition of GSK-3beta activity with the GSK3beta inhibitors SB-216763 and AR-A014418 controled cancer cell survival and proliferation, in a manner unrelated to Wnt/beta-catenin signaling and Akt activation, through decreasing telomerase reverse transcriptase (hTERT) expression and telomerase activity [256]. GSK-3beta inhibitors, lithium and SB-415286, have been also reported to overcome the inherent resistance of human hepatoma cells towards TRAIL-induced apoptosis. The inhibitors exerted their anticancer activity through a complex mechanism which involved the promotion of apoptotic signals (caspase-8, caspase-3 and TP53 activation) and of protective signals (JNK activation) [257].

## Wnt/GSK-3 Regulation of mTORC1 Pathway

Wnt signaling can regulate the mTORC1 pathway via GSK-3 by a beta-catenin-independent mechanism [189]. GSK-3 can phosphorylate TSC2 after it has been phosphorylated (priming phosphorylation) by AMPK. This would lead to inhibition of mTORC1. In contrast, Wnt signaling suppresses GSK-3-mediated phosphorylation of TSC2 and the mTORC1 pathway is activated. Inhibition of Wnt-mediated activation of mTORC1 by rapamycin suppressed Wnt-mediated cellular proliferation. Activation of AMPK by metformin suppressed Wnt-mediated cellular proliferation [192]. In addition to GSK-3, Dickkopf-related protein 1 (DKK1), Dvl and Axin also modulate the effects Wnt has on regulation of mTORC1 and cellular proliferation.

## Wnt/GSK-3 Signaling in the Regulation of SMAD1

Mothers against decapentaplegic homolog 1 (SMAD1) is a mediator of the transforming growth factor-beta (TGF-beta)/bone morphogenetic protein (BMP) superfamily of ligands. Both MAPK and GSK-3 were determined to phosphorylate SMAD1 on its linker region which resulted in its polyubiquitinylation and transport to the centrosome where it was degraded by proteasomes. The priming phosphorylation mediated by MAPKs (ERK, JNK, p38^MAPK^) to phosphorylate SMAD1 was most likely required for GSK-3 activity [258]. Wnt signaling decreased GSK-3 phosphorylation of SMAD1. Thus, the Wnt/GSK3/SMAD1 pathway plays an important role in embryonic pattern formation.

## Overview of Hedgehog Pathway

**Figure 7:**
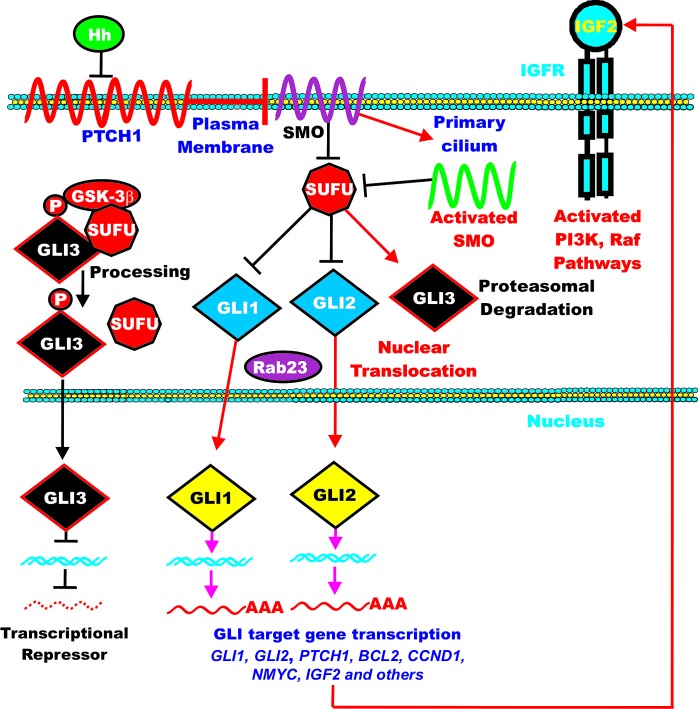
Overview of Hedgehog Signaling Pathway The Hh pathway normally inhibits the membrane spanning PTCH1 GPCR receptor which is indicated by a squiggly red line crossing the membrane 14 times. PTCH1 normally inhibits SMO which is indicated by a purple squiggly line which crosses the membrane 7 times. SMO is then activated in the primary cilium and serves to inhibit SUFU which is indicated by a red octagon. SUFU normally inhibits the GLI1 and GLI2 transcriptions factors which are indicated in blue diamonds. The Rab23 GTPase may aid in the nuclear translocation of the transcription factors and is indicated by a purple oval. In contrast, GLI3 is activated by SUFU and may serve as a transcriptional repressor and is regulated by GSK-3. It is indicated in a black diamond. One of the genes that are regulated by GLI signaling is *IGF2*. Dysregulation of Hh signaling pathway occurs in certain cancers. Resistance to Hh pathway inhibitors may result from mutations in the pathways which lead to increased pathway expression and autocrine IGF2 expression. This figure is presented to give the reader an idea of the negative regulation of the Hh pathway by various components

The hedgehog (Hh) pathway is an important developmental pathway that controls segmental pattern formation. There are three Hh genes, desert hedgehog (DHH), Indian hedgehog (IHH) and sonic hedgehog (SHH). These hedgehogs function as ligands for the 12-pass transmembrane receptor patched (PTCH1) which controls many developmental signaling events [259]. Hhs can function in some cases as mitogens, while in other cases they promote differentiation. An overview of the Hh pathway is presented in Figure 7.

Hh ligands bind their receptor PTCH1. This causes its internalization and degradation and release of Smoothened (SMO), a GPCR. SMO then promotes the dissociation of a suppressor of fused (SUFU)-glioma-associated oncogene homologue (GLI) complex. This allows the transcription factors GLI1 and GLI2 to translocate to the nucleus and ultimately induce the transcription of certain genes. In contrast, GLI3 is degraded during this process as it normally functions as a repressor.

GLI3 can be phosphorylated by PKA, GSK-3 and CK1 which results in GLI3 becoming a transcriptional repressor. Activated GLI proteins stimulate the transcription of Hh target genes, including: *GLI1*, *GLI2*, *PTCH1*, *CCND1*, *IGF2*, *MYC*, and *BCL2*. IGF-2 has been determined in some studies to subsequently induce the PI3K/PTEN/Akt/mTOR pathway and influence proliferation and survival as well as inhibit GSK-3beta [260].

The Hh pathway interacts with other signaling pathway regulators such as BMPs, parthyroid hormone and retinoids. The Hh pathway is aberrantly regulated in certain tumors. There is substantial interest in isolating inhibitors which can suppress its activity in various cancers and other disorders.

## GSK-3 Regulation of Hh Signaling

The Hh pathway is regulated by GSK-3 [261]. The Hh pathway is inhibited when GSK-3 is suppressed. GSK-3beta is a specific binding partner of SUFU, a negative regulator of the Hh pathway. SUFU suppresses GLI activity by sequestering GLI in the cytoplasm [262]. When GSK-3 phosphorylates SUFU, Hh pathway activity is increased. Hh pathway activation stimulates GSK-3beta to phosphorylate SUFU which induces the dissociation of SUFU from GLI3. GSK-3beta regulates the Hh pathway by phosphorylating GLI [263]. The Hh pathway activates the GLI transcription factor by suppressing the function of SUFU. SUFU recruits GSK-3beta into a trimolecular complex of GLI3/SUFU/GSK-3beta. GSK-3beta then phosphorylates GLI3 which results in a SUFU/GLI3 transcriptional repressor. Shh then dissociates the GLI3/SUFU/GSK-3 beta complex from GLI3 which prevents GLI3 processing and results in GLI3 becoming a transcriptional activator instead of a transcriptional repressor.

## Overview of Notch Signaling Pathway

Notch signaling is important in many processes including cellular fate decisions [264]. Notch signaling is activated by cell to cell interactions which result in the step wise cleavage of Notch first by a TACE- metalloprotease, and then a gamma-secretase [265-267]. This cleavage results in the release of the Notch intracellular domain (NICD). NICD can then translocate into the nucleus to bind transcription factors such as centromere binding factor (CBF1) which further recruits transcriptional co-activators including MAML1, histone acetyl transferases and cAMP response element-binding protein (CREB)-binding protein/p300 [268,269].

## GSK-3 and Notch Signaling Pathway

Notch signaling pathway is regulated by GSK-3. GSK-3beta phosphorylates the NICD. This phosphorylation event up-regulates NICD transcriptional activity by preventing its proteasomal degradation [75]. In contrast to the effects of GSK-3, Akt negatively regulates NICD activity [270]. GSK-3beta phosphorylates domains in the NICD of Notch1 (S/T-P-S/T) which are important for the nuclear localization of NICD [271]. Phosphorylation of these domains by GSK-3beta increased the transcriptional activity of NICD. In contrast, other studies have shown that GSK-3 phosphorylation can decrease Notch1 protein levels and its transcriptional activity [272]. beta-catenin could also modulate the transcriptional activity of Notch1/NICD [273]. Beta-catenin levels are usually increased when GSK-3 is suppressed. Other studies have indicated that GSK-3beta phosphorylation of Notch downregulates Notch activity [274]. Some of these differences in the effects of GSK-3 on the phosphorylation of Notch observed in the different studies may be related to the different cell lines utilized or culture conditions. Different culture conditions or cell lines containing different mutated activated oncogenes activated and tumor suppressor genes inactivatedmay affect Notch activity. These studies and others indicate the complex interactions between the GSK-3, Wnt/beta-catenin and Notch signaling pathways.

## GSK-3 Inhibitors

One of the first described inhibitors of GSK-3 is lithium [275]. Lithium carbonate has been used to treat mania patients since 1871. It was approved by the FDA in 1970 and it is currently used to treat many different types of patients with depressive disorders [276]. Early clinical studies indicated that lithium had effects on HSCs and other hematopoietic cells [277-279]. Lithium increased circulating HSCs and peripheral blood counts [280]. In 1996, it was proposed that lithium could mobilize HSCs for BM transplantations [281]. An important consequence of suppression of GSK-3 is stimulation of both the Wnt/beta-catenin and PI3K/PTEN/Akt/mTORC1 pathways. However, GSK-3 has many biological effects [275,282,289-299].

More than 50 GSK-3 inhibitors have been described including: lithium [300], AR-A014418 (beta, IC_50_ =104 nM) [301], AZD1080 (alpha & beta, orally active, brain permeable, with K_i_s of 6.9 nM and 31 nM respectively) [302], AZD2858 (alpha & beta IC_50_s of 68 nM) [303], BIO (alpha & beta IC50 of 5 nM) [304], CHIR-99021 (alpha & beta, IC_50_s of 10 nM/6.7 nM) [305], CHIR-98014 (alpha & beta, IC_50_ of 0.65) [306], LY2090314 (alpha & beta, IC_50_s of 1.5 nM and 0.9 nM respectively) [307], SB-216763 (alpha & beta, IC_50_s of 34.3 nM) [308, 309], SB-415286 (alpha & beta IC_50_s of 78 nM and 31 nM respectively) [310, 311], TDZD-8 (beta, IC_50_ of 2 microM [312] and TWS119 (beta, IC_50_s of 30 nM) [313]. Most GSK-3 inhibitors are ATP-competitive and do not differentiate between either GSK-3alpha or GSK-3beta [28]. Tideglusib (NP031112) is an irreversible, non ATP-competitive GSK-3beta inhibitor with IC_50_ of 60 nM, which is in clinical trials [284, 314]. Table 1 present a listing of some GSK-3 inhibitors and their structures and references.

**Table 1:** GSK-3 Inhibitors

	Inhibition	Inhibition		
Inhibitor Name	GSK-3α	GSK-3β	Structure	Ref/Clinical Trials
SB216763	+	+	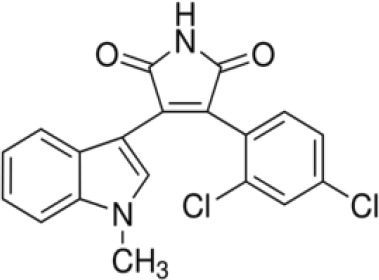	[308, 309]
TWS119		+	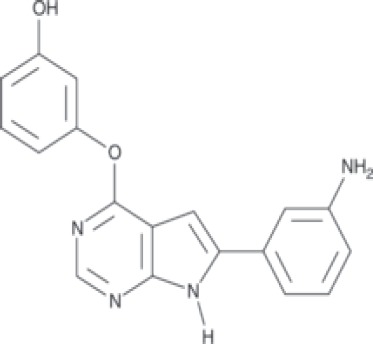	[312]
SB415286	+	+	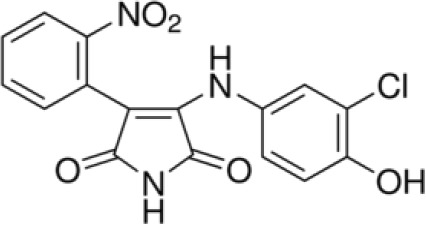	[310, 311]
Tideglusib (NP031112, NP-12)		+	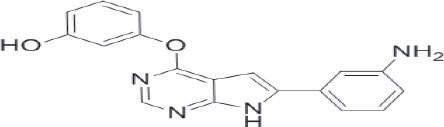	[284, 314] NCT00948259 NCT01049399
AR-A014418		+	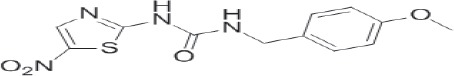	[301]
TDZD-8 (NP01139)		+	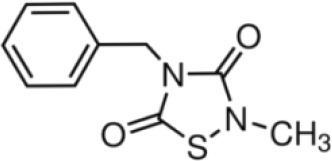	[302]
LY2090314	+	+	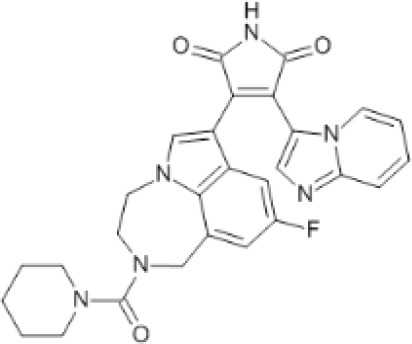	[307] NCT01632306 NCT01287520 NCT01214603

Clinical Trials Phase I/II: LY2090314 works in combination with different chemotherapies in treating participants with metastatic pancreatic cancer ClinicalTrials.gov Identifier: NCT01632306 Recruiting Phase I: LY2090314 in combination with pemetrexed and carboplatin in patients with advanced/metastatic cancer ClinicalTrials.gov Identifier: NCT01287520 Completed Phase 2 study of intravenous LY2090314 in participants with acute leukemia ClinicalTrials.gov Identifier: NCT01214603 Completed

## Clinical Trials with GSK-3 Inhibitors

Lithium carbonate has been in clinical trials with bipolar disorder patients [300]. It is also involved in additional clinical trials for patients with Alzheimer disease (NCT01055392 & NCT00088387), bipolar disorder (NCT01543724, NCT00870311), spinocerebellar ataxia type 3 (NCT01096082) and Machado Joseph disease. Tideglusib has been in clinical trials for patients with Alzheimer's disease (NCT00948259) and progressive supranuclear palsy (NCT01049399). Divalproex (Valproate semisodium) is used for treatment of patients suffering from bipolar disorder, epilepsy and migraines. Divalproex has been in clinical trials in combination with lithium, for patients with Alzheimer's disease (NCT00088387), Valproic acid (VPA) is an anticonvulsant and mood-stabilizing drug which can inhibit GSK-3beta. It has been examined in clinical trials (NCT01548066) to determine whether it will prevent hair loss. It has also been examined in a clinical trial (NCT00385710) to determine if it can aid patients with progressive supranuclear palsy. LY2090314 has been in clinical trials for metastatic pancreatic cancer [NCT01632306], advanced or metastatic cancer in combination with pemetrexed and carboplatin [NCT01287520] and acute leukemia [NCT01214603].

## GSK-3 Inhibitors in Pre-Clinical Anti-Cancer Studies

GSK-3 inhibitors may eventually be used in the treatment of certain cancers. GSK-3 is believed to exert pro-proliferative effects in solid cancers including: CRC, glioblastoma, pancreatic cancer and ovarian cancer and in blood cancers including MM, ALL, AML and CLL. Therefore it may be appropriate to treat these cancers and potentially others with GSK-3 inhibitors [124,284-288]. Treatment of the KG1a, K562 and CMK leukemia cell lines with the GSK-3 inhibitor SB-415286 resulted in GSK-3beta S9 phosphorylation beta-catenin stabilization, cyclin B downregulation, cell growth inhibition, cell cycle arrest at G_2_/M and apoptosis [289]. Blocking the death receptor pathway with an inhibitor of caspase 8 did not neutralize the effects of the GSK-3 inhibitor. The GSK-3 inhibitor treatment resulted in dephosphorylation of Bcl-2, downregulation of Bcl-X_L_ and led to the depolarization of the mitochondrial membrane potential. The role of stabilized beta-catenin induced by the treatment of the leukemia cells with the GSK-3 inhibitor in the induction of apoptosis of these cells is not clear. The GSK-3 inhibitors SB-415286 and lithium as well as arsenic trioxide inactivated GSK-3beta in AML (APL) cells by inducing the phosphorylation of S9 on GSK-3beta [290]. However, in other cancer types such as breast cancer and medulloblastoma, increased GSK-3 expression is associated with the induction of apoptosis [291,292]. It remains controversial as to whether suppressing GSK-3 activity with small molecule inhibitors will inhibit or promote cancer growth.

1i is a selective ATP competitive inhibitor of GSK-3. 6-bromoindirubin-3-oxime (BIO) and TWS119 are also GSK-3 inhibitors. BIO inhibited GSK-3 and activated Wnt/beta catenin signaling, thereby maintaining the pluripotency of human and mouse ESCs [208]. Continued GSK-3 inhibition or Wnt pathway activation promoted ESC differentiation into multipotent mesendodermal progenitors or their differential progenitors [293-295]. As stated previously, GSK-3 interacts with the Wnt, Hh and Notch pathways. These interactions can under the appropriate conditions regulate cell fate determination and maintenance of stem cells. GSK-3 can phosphorylate the GLI2 transcription factor, which can effect differentiation [296]. The self-renewal of ESCs was enhanced when they were cultured with leukemia inhibitory factor (LIF) and BMP3 and GSK-3 were suppressed. The mechanism responsible for the enhanced self-renewal is not clear. It was proposed that the expression of pluripotency factors such as Oct-4 and Nanog was prevented in these conditions [295]. GSK-3 may phosphorylate and inactivate other transcription factors associated with pluripotency, such as c-Myc and c-Jun. 48 derivatives of the GSK-3 inhibitor 1i were synthesized by Bone and colleagues. The abilities of these GSK-3 inhibitors to maintain undifferentiated ESCs were determined. The effectiveness of these compounds on maintaining undifferentiated ESCs was linked with inhibition of GSK-3 [297]. Thus GSK-3 inhibitors may be used to both maintain stem cell pluripotency as well as to expand these cells. These inhibitors may also be effective with induced pluripotent stem cell generation (iPSC) [295].

GSK-3 inhibitors may be appropriate for the treatment of certain diseases including: diabetes, obesity, ischemia, cancer, sepsis, colitis and neurological disorders. However, the evaluation of GSK-3 inhibitors in clinical trials has been hampered by the fear that inhibition of GSK-3 may stimulate or aid in malignant transformation as GSK-3 can phosphorylate such pro-oncogenic factors as beta-catenin, c-Jun and c-Myc which targets them for degradation [28]. However, no studies have been reported suggesting that treatment of mice with GSK-3 inhibitors resulted in an increase in cancer incidence. In contrast, studies in prostate, pancreatic, ovarian, thyroid, melanoma, brain, pheochromocytoma, paraganglioma, colorectal, and hematopoietic cancers have indicated that GSK-3 inhibitors could suppress proliferation [298]. In fact, many patients with bi-polar disorder have been treated with lithium for prolonged periods of time. There does not appear to be any evidence that these patients have increased incidences of cancer. In the studies by Wang *et al*, treatment on mice with mixed-lineage leukemia (MLL) like leukemia (see below) with lithium actually increased the survival of the mice approximately 50%.

Treatment of human leukemias containing *MLL* translocations with GSK-3 inhibitors GSK-3 IX, SB216763, and allsterpaullone inhibited their growth of [64]. GSK-3beta activity was determined to be essential for the maintenance of *MLL* LSC. However GSK-3beta activity was not necessary for normal myeloid progenitors or progenitors transfected with other non-*MLL* fusions. GSK-3alpha and GSK-3beta performed similar functions in regulating the growth of *MLL*-transformed leukemias. p27^Kip-1^ was critical for the effectiveness of the GSK-3 inhibitors. Knock-down of p27^Kip-1^ prevented the growth arrest of the GSK-3 inhibitors specifically in *MLL*-transformed cells but not non-*MLL* fusion protein-transformed cells. GSK-3 is believed to support the maintenance of *MLL*-transformed leukemic cells by promoting p27^Kip-1^ degradation. Upon treatment of the *MLL*-transformed cells with GSK-3 inhibitors, p27^Kip-1^ accumulated specifically in the *MLL*-transformed cells and cell cycle arrest occurred.

## Effects of Certain Small Molecule Inhibitors on GSK-3 Activity

Certain small molecule inhibitors synergized with GSK-3beta inhibition to result in cell death [299]. The multi-kinase inhibitor sorafenib induced GSK-3beta which actually provides a survival signal in melanoma cells. When a constitutively-active form of *GSK3B* was introduced into the melanoma cells, elevated levels of anti-apoptotic Bcl-2, Bcl-X_L_ and survivin were detected while decreased levels of pro-apoptotic Noxa were observed. Elimination of GSK-3beta activity increased the activity of sorafenib.

## Summary

Many different biological processes are regulated by GSK-3 alpha and beta. GSK-3 interacts with many signaling pathways. Critical pathways often dysregulated in cancer are the PI3K/PTEN/Akt/mTORC1 and Wnt/beta-catenin pathways. GSK-3 interacts with both Akt, TSC2 to regulate mTORC1. Akt has a negative effect on GSK-3 as when GSK-3 is phosphorylated by Akt, GSK-3 can not phosphorylate TSC2. This has a pro-proliferative effect as mTORC1 is not suppressed by TSC2. GSK-3 has a negative effect on mTORC as it phosphorylates and promotes the activity of TSC2 which inhibits mTORC1. This has an anti-proliferative effect. mTORC1 and GSK-3 also interact to regulate p70S6K which can indirectly regulate Akt. GSK-3 interacts with the Wnt/beta-catenin pathway at multiple levels; in some circumstances it inhibits the activity of Wnt/beta-catenin pathway by phosphorylation of beta-catanin which results in its proteasomal degradation, while in other cases it promotes the pathway by phosphorylating LRP5/6 which results in the stabilization of beta-catenin. This latter effect is especially prominent in certain CIC, where GSK-3 inhibitors may prove clinically effective; however, they probably will have to be administered with beta-catenin or downstream TCF/LEF inhibitors. GSK-3 also interacts with the Raf/MEK/ERK, Hh, Notch, and other important regulatory pathways. It is intriguing to consider why inhibiting GSK-3 does not have dire consequences as so many pathways will be affected which will lead to activation/suppression of various signaling pathways. However, the GSK-3 inhibitor lithium has been used to treat patients for many years now without apparent increases in the cancer incidence. GSK-3 is a kinase with many biochemical, biological and neurological effects. Understanding of these effects may lead to improved therapies for certain diseases and conditions.
